# Catheter ablation of ventricular tachycardia: strategies to improve outcomes

**DOI:** 10.3389/fcvm.2023.966634

**Published:** 2023-08-14

**Authors:** Muthiah Subramanian, Auras R. Atreya, Daljeet Kaur Saggu, Sachin Yalagudri, Narasimhan Calambur

**Affiliations:** ^1^ Department of Cardiology, AIG Institute of Cardiac Sciences, Gachibowli, India; ^2^ Department of Cardiology, University of Arkansas Medical Sciences, Little Rock, AR, United States

**Keywords:** catheter ablation, electro-anatomic mapping, ventricular tachycardia, improving VT ablation, substrate mapping

## Abstract

Catheter ablation of ventricular arrhythmias has evolved considerably since it was first described more than 3 decades ago. Advancements in understanding the underlying substrate, utilizing pre-procedural imaging, and evolving ablation techniques have improved the outcomes of catheter ablation. Ensuring safety and efficacy during catheter ablation requires adequate planning, including analysis of the 12 lead ECG and appropriate pre-procedural imaging. Defining the underlying arrhythmogenic substrate and disease eitology allow for the developed of tailored ablation strategies, especially for patients with non-ischemic cardiomyopathies. During ablation, the type of anesthesia can affect VT induction, the quality of the electro-anatomic map, and the stability of the catheter during ablation. For high risk patients, appropriate selection of hemodynamic support can increase the success of VT ablation. For patients in whom VT is hemodynamically unstable or difficult to induce, substrate modification strategies can aid in safe and successful ablation. Recently, there has been an several advancements in substrate mapping strategies that can be used to identify and differentiate local late potentials. The incorporation of high-definition mapping and contact-sense technologies have both had incremental benefits on the success of ablation procedures. It is crucial to harness newer technology and ablation strategies with the highest level of peri-procedural safety to achieve optimal long-term outcomes in patients undergoing VT ablation.

## Introduction

Catheter ablation has an important role in the management of patients with ventricular tachycardia (VT) (1, 2). Advances in pre-procedural imaging, intra-procedural mapping, and ablation techniques have improved the outcome of catheter ablation. As catheter ablation becomes a first-line therapy, it is crucial to employ a systematic approach for clinical assessment, mapping, and ablation of VT. The goals of ablation includes (a) elimination or reduction of the underlying arrhythmia, (b) maintaining patient safety and (c) limiting collateral injury to improve long term outcomes. In this review we will address the importance of understanding the underlying substrate, pre-procedural planning, and strategies to improve mapping and ablation of VT.

## Pre-procedural planning

### Understanding the substrate

A careful evaluation of the patient history, 12 lead ECG, and imaging are critical for understanding the mechanism and localization of the origin of VT. Anatomic substrate, electrophysiological characteristics and approach to catheter ablation are different among patients with ischemic and non-ischemic cardiomyopathies. While the scar in ischemic cardiomyopathy is usually sub-endocardial, patients with non-ischemic cardiomyopathy (NICM) often have patchy VT circuits located in mid-myocardium and epicardial location (3, 4). Patients with non-endocardial scars may require epicardial access, bipolar ablation, and more advanced ablation techniques (5). Patients with NICM, who have failed prior endocardial ablations, often have more extensive scar in the epicardium with more than 50% of these patients requiring an epicardial ablation (6, 7). In addition, these substrates can progress, especially in conditions like sarcoidosis, hypertrophic cardiomyopathy, and arrhythomgenic right ventricular dysplasia (8). Treatment of the underlying disease is important to prevent disease progression, recurrence of arrhythmias and heart failure hospitalizations.

### ECG characterization

Evaluation of the ECG in sinus rhythm and VT (if available) can help characterize the site and extent of the underlying scar. Presence of q waves in sinus rhythm can provide a clue regarding the location of myocardial scarring. In patients with NICM, fragmented QRS complexes can be used to aid scar localization (9). The 12 lead ECG allows for localization of the “exit site” of VTs with a predictive accuracy of >70% to guide initial mapping efforts (10). Utilizing the 4-quadrant approach allows for easy localization of cardiac structures where the VA may exit from. It is important to understand that depending on the size of the circuit, this exit site may be up to 2–5 cm^2^ away from the critical isthmus (10). Clinical and automated VT-ECG algorithms have been developed to predict SOO, but its use remains limited (11, 12). Several algorithms are available for identification of potential epicardial VT exits, although they are less useful in patients with extensive scar (13, 14). Irregular cycle lengths and pleomorphism of underlying VT morphology are potential indicators of underlying myocardial inflammation (15). Electrocardiographic imaging (ECGI) is a non-invasive mapping strategy that combines a computed tomography scan of the chest and with a continuous 256-lead ECG recorded by a multi-electrode vest to accurately predict the VA exit site (16).

### Pre-procedural imaging

Pre-procedural imaging can be very useful in guiding the operator towards the areas of interest and increasing success rate of ablation. Contrast enhanced MRI (CMR) has become the cornerstone of pre-procedural imaging because of its ability to delineate myocardial scar (17, 18). In patients with idiopathic PVCs or VT, CMR can visualize areas of scarring that may have not identified on routine echocardiography. Most importantly, CMR can help guide electro-anatomic mapping and delineate areas of potential substrate for ablation. Along with ECG, CMR can be used to determine the predominant scar pattern and likelihood of mid-myocardial and epicardial ablation targets (19). Beyond the need for up-front epicardial access, CMR can also be used to identify the critical site for VT circuits based on the signal intensity and transmurality of the scar (20, 21). Delayed gadalonium enhancement, and grey border zones zones have good correlation with conducting channels and can identify critical isthmus sites in >70% of cases (22). However artifacts related to ICD lead remain one of the major limitations in interpretation of images in patients with cardiac implantable electronic devices (CIEDs). Recent studies have tested the feasibility of wideband inversion recovery CMR protocols in patients with CIEDs (23, 24). These sequences significantly reduced hyperenhancement artifacts, offering a potential solution for patients with ICDs.

**Figure 1:**
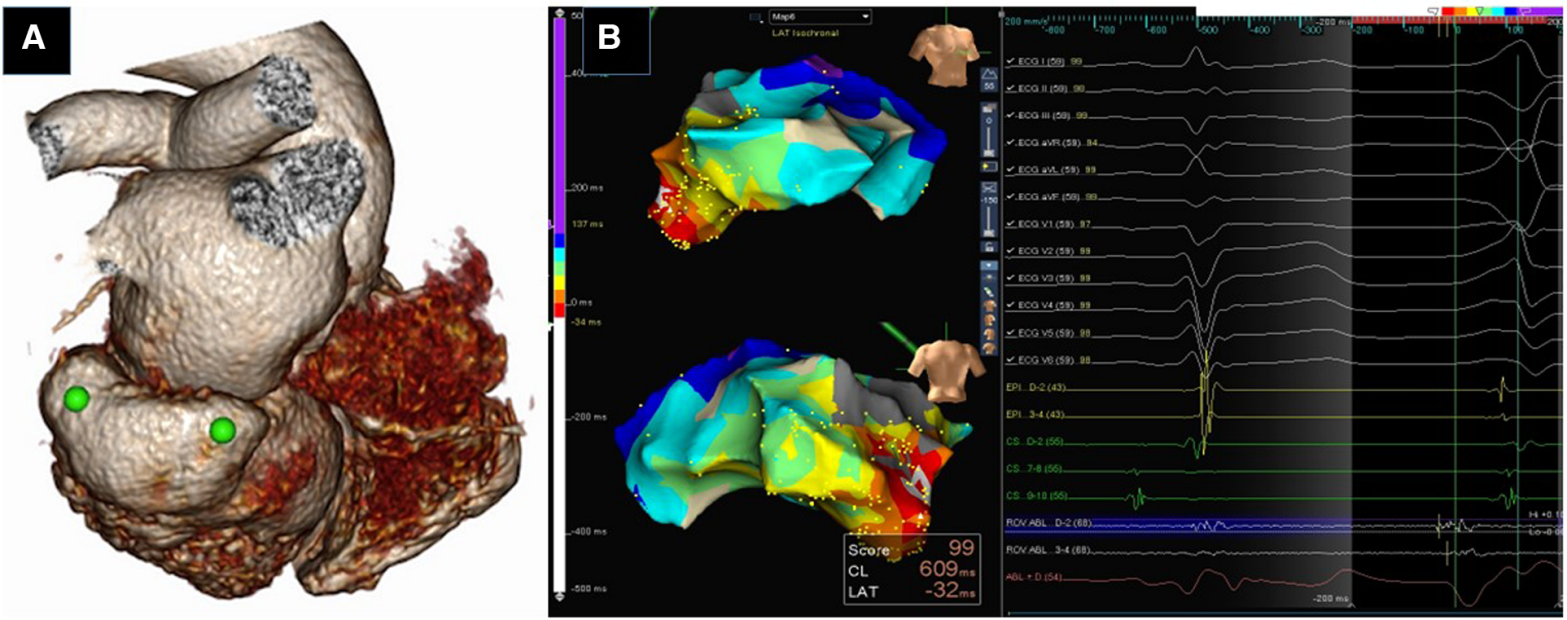
Utility of Pre-procedural imaging. Three dimensional volume rendered CT image of heart showing submitral posterobasal aneurysm (green dots) (**A**). 3D Electroanatomic Images in RAO and PA showing earliest activation in the aneurysm (white arrow) (**B**). RFA at this site eliminated the PVCs.

Computed tomography is a viable option when MRI is not feasible. It has higher spatial resolution than CMR and its use is increasing as a pre-procedural tool during VT ablation (Figure 1). However, it is limited by a lower contrast-to-noise ratio within myocardial tissue, contributing to inferior scar delineation (25). CECT combined with dynamic, perfusion imaging has been shown to accurately characterize LV scar and border-zone substrates (26). Recent studies have evaluated the role of CT imaging (with post-processing using proprietary MUSIC software, IHU LIRYC Bordeaux and Inria Sophia Antipolis, France), in identifying ridges/channels which denote preserved myocardial tissue surrounded by scar (thinned out areas) that could potentially be targets for ablation (27, 28). Cardiac anatomy and substrate characterization identified by CMR and CT imaging can be integrated into the EAM systems for guidance during the ablation procedure (21, 29). With this technique, areas of scar identified on CT imaging correlate well with low voltage areas on the EAM system (both unipolar and bipolar voltage maps) (30, 31).

**Figure 2:**
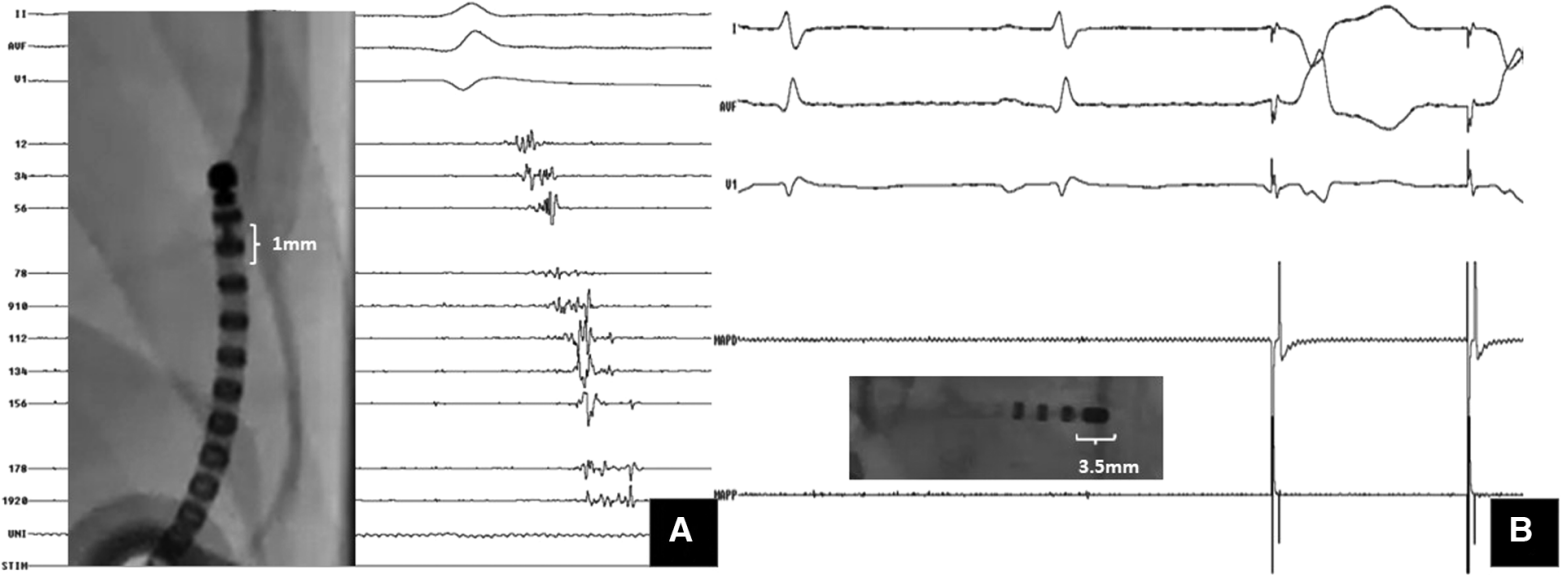
Importance of multielectrode mapping. A Case of ARVC with VT storm: substrate mapping with (**A**) multipolar catheter (1 mm electrode size) showed isolated late potentials within the scar; (**B**) mapping with the ablation catheter (3.5 mm tip electrode) at the same site could not pick up these signals. However, pacing at same site captured with latency.

In patients with acute inflammatory cardiomyopathies, a fluorodeoxyglucose-positron emission tomography (FDG-PET) scan have an incremental advantage in diagnosis and assessing the extent of inflammatory burden. In patients with inflammatory cardiomyopathies like sarcoidosis, the results of CA during the inflammatory phase are poor and the recurrence rates were higher (32). Combining FDG PET with CMR allows for better characterization of scar burden and myocardial inflammation (33).

Lastly, cardiac computed tomography (CT) can also provide an accurate roadmap of anatomy and to minimize complications. Major collateral injury to adjacent structures like the heart, liver and coronary vessels have been reported in 4%–10% of patients during subxiphoid percutaneous epicardial access (34, 35). We recently used a pre-procedural CT imaging to plan epicardial access in patients undergoing VT ablation. This allows the operator to pre-determine the ideal site, angle, and trajectory of needle insertion to avoid collateral injury (36). Prior knowledge of the needle course can aid in increasing the safety of epicardial access, especially in patients with a distorted anatomy.

## Strategies for vascular access, anesthesia, and hemodynamic support

### Safe vascular access and approach

Vascular complications due to access site issues are to be strictly avoided. Complications are due to multiple large-bore access sites followed by intra-procedural systemic anticoagulation. Ultrasound guidance has been shown to improve the safety by reducing vascular access-related complications (37). Ultrasound guided femoral puncture has a short learning curve and does not interfere with the normal workflow of EP procedures. Meta-analysis of observational trials have showed up to a 65% reduction in major and minor vascular complications using ultrasound guidance for femoral vein access in EP procedures (38). Patients with both aortic and mitral mechanical valves present a challenge to achieve LV endocardial access. In these rare circumstances, both a transapical access and inter-ventricular septal access have been described. A detailed description of the epicardial access technique and challenges is beyond the scope of this manuscript.

### Anesthetic considerations

Discussions between the electrophysiologist and anesthesiologist prior to procedure can be extremely useful in helpful in increasing the success rate of CA and mitigating complications. Depending on the patient and the operator, VT ablation is usually performed under general anesthesia or under monitored anesthesia care (MAC). Advantages of MAC with sedation are avoiding anesthetics that can potentially depress myocardial contractility and decrease the inducibility of VT (39). Idiopathic PVCs may be difficult to induce, and it is better to avoid intravenous anesthetics that reduce sympathetic tone (dexmedetomidine, midazolam, and propofol) in these patients. For patients undergoing prolonged, complex procedures who have unstable rhythms or poor cardiopulmonary reserve, GA is preferred. Controlling patient ventilation during GA aids in maintaining a constant tidal volume and I:E (Inspiratory:expiratory) ratios. This can help to improve the quality of the electro-anatomical map as well. Controlled apnea during the procedure can be a useful strategy to improve catheter contact during ablation. In addition, an end inspiratory breath hold can also provide a safer needle trajectory during epicardial access, by minimizing collateral damage to adjacent structures (36). Neuromuscular blockade agents should be avoided when the planned ablation site is closer to the phrenic nerve, as they can prevent phrenic nerve localization by pacing (39).

### Hemodynamic support

In recent years, mechanical circulatory support has be used as both an emergent “rescue” and prophylactic therapy for high-risk patients undergoing VT ablation. Acute hemodynamic compromise can occur not only during sustained VT, but also during sinus rhythm or right ventricular pacing. The PAAINESD score is a risk stratification tool that has been used to guide selection of patients for mechanical circulatory support (MCS) (40). The advantages of MCS are mapping of a greater number of VTs using activation and entrainment mapping in a sicker cohort of patients. Intra-aortic balloon pumps, TandemHeart, Impella axial blood flow pump, and extracorporeal membrane oxygenation have all been used in VT ablation. The major advantages of percutaneous ventricular assist devices, compared to IABP, is that patients maintain end-organ perfusion during VT for longer periods (41). The decision to use MCS during VT ablation needs to be individualized. Integrated care between cardiologists, surgeons, and anesthetists is important for planning vascular access, optimizing hemodynamics during VT ablation.

## Role of intra-procedural imaging

Intra-procedural imaging for VT ablation involves two major aspects—image integration into the electro-anatomic mapping (EAM) system and real-time imaging during ablation. Integrating CMR images with EAM can enhance the scar definition and has the potential to facilitate targeted approaches to VT ablation. When patients undergoing MRI-derived scar-guided ablation were compared to those undergoing traditional ablation, there is a lower incidence of recurrence in the former (29, 42). Integrating CT imaging can also be an useful strategy as it can delineate epicardial fat, coronary anatomy, and the course of the phrenic vessels (25, 30). Intra-cardiac echocardiography is commonly used during VT ablation. It is an useful tool to understand catheter contact when mapping intracavitary structures such as papillary muscles and moderator band (43). In addition it can serve as an important safety tool for asses pericardial effusion and assessing ablation lesion depth (44). Real-time CMR guided ablation procedures have been described in a few case series (45). The advantages of this system is ability to image and assess the entire myocardium and ablation lesions in real time. Real-time CMR system may substantially change the work-flow during VT ablation.

## Optimizing anticoagulation during ablation

Anticoagulation strategies during VT ablation are dependent on right vs. left sided ablation and whether the patient was already using a vitamin K antagonist or direct oral anticoagulant prior to the procedure. During ablation of left sided VT, therapeutic anticoagulation is crucial and special considerations are needed depending on the LV access route. During LV endocardial ablations, full dose heparin is generally given once trans-septal access is achieved. An initial bolus of 100 U/kg followed by intermittent boluses or continuous infusion of heparin to main an ACT >300s is ideal (46). It is important to have a continuous flush of the side arm of the access (transeptal) sheath with heparinized saline.

## Mapping strategies

### Mapping during sinus rhythm: creating the substrate map

For patients with VTs that are difficult to induce or hemodynamically unstable, activation and entrainment mapping cannot be performed. Substrate-mapping strategies have a crucial role and are required for successful VT ablation. Most VT exit sites are in the periphery of the scar (i.e., borderzone), while the critical isthmus resides within the dense scar. In the endocardium, bipolar peak-to-peak voltage definitions are used to describe preserved tissue (>1.5 mV), borderzone tissue (0.5–1.5 mV) and dense scar (<0.5 mV) (47). The limitations of relying on bipolar peak-to-peak electrograms are that it can be affected by several biophysical determinants, including mapping bipole electrode geometry and orientation. Dichotomizing cardiac tissues into scar vs. healthy tissues based on single bipolar voltage cut offs can be misleading. Hence, ablation based on the geography of low bipolar voltage based scar is generally avoided (48, 49). Several authors have described that areas of relatively higher voltage can be within the scar. These areas correspond to surviving bundles of myocardial cells within the scar tissue, functioning as conducting channels. By applying a stepwise reduction in the definition of abnormal voltage from 0.5 to 0.1 mV, it was found that most conducting channels have voltage scar definitions of <0.2 mV (50, 51). By adjusting the bipolar voltage limits, these voltage channels can be identified in greater than 85% of patients with mappable ischemic VT (52). However, the specificity of these channels to predict the location of the VT isthmus is only 30%. In the presence of normal endocardial bipolar voltage, a low tissue unipolar voltage (<8.3 mV for LV and <5.5 mV for RV free wall) is considered a sensitive marker of myocardial disease in the intramural and epicardial tissues (53, 54). However, these strategies are unable to reliably identify whether this residual excitable substrate is in the epicardial or mid-myocardial layers. Recently, Qian and colleagues found that endocardial unipolar voltages were significantly higher in sites with deep intramural excitable substrates compared with transmural scars (55).

Fractionated electrograms are considered indicative of preserved myocardial fibers interspersed within a fibrous scar tissue. The heterogeneities in the cellular electric properties and local tissue architecture create regions of discontinuous slow conduction (56). Due to reduced intercellular coupling, irregularities in depolarization and propagation in adjacent cells become separated in time, causing distinct fractionated deflections in the local electrogram. Local Abnormal Ventricular Activities (LAVAs) refer to sharp high-frequency ventricular potentials occurring after far-field electrograms and display double or multiple high frequency signals (57). They are generated by poorly coupled viable fibers within the scar. Jais and colleagued coined this term to qualify multicomponent electrograms that can be buried in a broader far-field electrogram. Complete elimination of LAVA was associated with a superior survival free from recurrent VT during follow up (57).

Late potentials are characterized by low amplitude, usually fractionated signals, with a bipolar amplitude <1.5 mV. They are detected after the local ventricular electrogram and QRS. These late potentials originate from slow-conducting surviving bundles of myocardium in areas of fibrotic scar tissue (58). Isolated late potentials are defined as the second or subsequent electrograms that are separated from the initial ventricular electrogram by an isoelectric interval (isolated component >20 ms after the end of the QRS) or very late potentials (isolated component >100 ms after the end of the surface QRS) (59). Electrograms during sinus rhythm in the delayed isochrones of activation, especially in regions of deceleration (crowding of isoschrones of activation) have been shown to be in anatomic proximity to the critical isthmuses of VT circuits (60). Iscochronal late activation mapping utilizes this principle to help target ablation to crowded isochrones (defined as color-coded zones encompassing >40 ms per 10 cm). Ideally, ILAMs are displayed as eight equally distributed isochrones of activation, which represent 12.5% of the window of activation. The creation of isochronal display of sinus rhythm activation within scar allows for visualization of both slow conduction and late activated regions (61, 62).

However, some sites yielding late potentials during sinus rhythm can be bystander sites during VT, despite being areas of slow conduction. Late potential mapping can have a very low sensitivity and moderate specificity for VT channels, especially in patients with a large scar burden. The presence of multipotential electrograms, long stimulus-QRS intervals during pace mapping, and the presence of late potentials inside voltage channels can increase the specificity of late potentials (63). Hence it may be prudent to combine identification of both abnormal voltage areas and abnormal local electrogram characteristics as optimal targets of VT ablation.

Substrate-based mapping strategies that target only late potentials may miss critical arrhythmogenic substrates, especially in early activating regions such as the septum. A source-sink mismatch or fibrotic barrier between up and downstream elements can produce a functional block within the scar (64). One of the strategies to increase identification of late potentials is to decouple the abnormal ventricular potentials from far-field ventricular potentials by changing the activation wavefront through differential pacing (65). Changing the pacing wavefront can unmask LAVA that were not obvious during sinus rhythm. Utilizing these strategies, LAVAs can be targeted during substrate mapping and subsequent ablation. The dynamic responses to differential pacing can vary among patients depending on their electrophysiological differences in scar related conduction abnormalities. The Physio-VT mapping model utilizes different responses of individual intracardiac electrograms to RV and LV pacing vs. sinus rhythm to improve VT substrate resolution and mapping (62).

Reduced cell-to-cell coupling due to altered distribution of gap junction proteins can contribute to slowing or conduction block at faster rates. This regional heterogeneity can be unmasked by programmed ventricular extra-stimulus pacing. This kind of pacing can potentially identify late potentials within scar or at scar border zones that are likely to participate in the initiation and maintenance of VT. Decremental evoked potentials (DEEPs) mapping utilizes a specific S1–S2 protocol to deliver extrastimulus mapping from the right ventricle. DEEP mapping has been found to be more specific than late potential mapping for identifying critical targets with in the diastolic pathway of VT (66).

Another strategy described by Di Biase and colleagues is scar homogenization. This usually involves ablation of the entire scar, with particular focus on late fractionated signals in the scar tissue (67). In this strategy, the authors targeted all endocardial and epicardial signals persisting longer than 70 ms or displaying 4 or more deflections or an amplitude less than 1.5 mV. While this strategy may eliminate VT, there is concern if extensive ablation may result in worsening LV function post procedure. This approach may be useful in patients with small scars and relatively preserved ventricular function.

Current substrate-based ablation strategies emphasize the need for high density mapping to identify and target all multicomponent electrograms. High density multi-electrode mapping has brought about a paradigm shift in substrate characterization during VT ablation. Local electrogram voltages are dependent on the recording electrode size, inter-electrode spacing, and direction of wavefront propagation (68). The use of multi-electrode mapping catheters with small (1.0 mm) closely spaced electrodes resulted in a 22% smaller low voltage area (<1.5 mV) and a 47% reduction in dense scar size (<0.5 mV) in animal models (69, 70). Another advantage of these high-density catheters is that they minimize the effects of far-field signals and aid in the identification of heterogeneity within low-voltage scars. Higher mapping densities are associated with a better endocardial LAVA identification and ablation outcomes.

Pace-mapping within scar can identify slow conduction areas by a long-stimulus to QRS interval latencies (>40 ms), that can correlated with VT isthmus sites (71). However, operators need to remember that a long stimulus-QRS interval can also occur in bystander regions. de Chillou and colleagues elegantly described the identification of the critical isthmus of the VT circuit by pace-mapping (72). A good pace map obtained from a scar border region can suggest an approximate location of the VT exit. More importantly, a discrepancy in QRS morphology between VT and pace-map does not necessarily imply the site is located far from the VT circuit. An abrupt transition from an area of perfect pace-mapping to an area of poor match (such as the entrance site) can correspond with the VT isthmus. During pace mapping it is important to remember that multiple exits can arise from scar and functional block can occur during VT, that may not be present in sinus rhythm.

### Mapping during ventricular tachycardia

Non-inducibility of the clinical tachycardia is a major limitation while attempting to map and ablate VT. Earlier reports indicate difficulty in induction of VT in approximately 25%–40% of patients of idiopathic left ventricular tachycardia (73). Using a systematic induction protocol along with the appropriate use of pharmacological agents resulted in a high induction rate of fascicular VT (74). In a subset of patients, sustained idiopathic left ventricular VT could only be induced by pacing from within the left ventricle. Isoproterenol enhances induction of sustained VT in up to 70% of patients without VT at baseline. In some patients, the administration of a low dose of a class 1A drug may enhance the slow conduction of specialized Purkinje fibers and facilitate induction of stable VTs. Nazer and colleagues found that an initial programmed electrical stimulation and entrainment mapping under conscious sedation was important for patients with NICM referred for epicardial ablation. In their series, empiric ablation of endocardial and epicardial scar would have missed the clinical VT in 20% of patients (75).

For those patients with inducible and hemodynamically tolerated VT, point by point activation mapping can be performed during VT focusing on the abnormal electrograms. Local electrograms that are activated during mid-diastole, especially those in the middle 25%–75% of diastole, should be targeted. Entrainment mapping remains one of the cornerstones of activation mapping strategies during stable VTs (76, 77). Local electrograms from the mapping catheter can exhibit a lot of artifacts during entrainment, making it difficult to interpret the electrograms. The use of adjacent electrodes can be a useful strategy when interpreting entrainment (78). In addition, using the *N* + 1 technique difference allows entrainment mapping to be used when the local electrogram from the pacing catheter has artefacts after high output pacing (79). Understanding the effect of variables such as pacing current strength, electrode size/spacing, filtering, and noise can help us avoid errors in interpretation during entrainment mapping.

Due to the incorporation of contact mapping technologies that utilize multielectrode acquisition, there is an improved understanding of activation within the myocardial wall during VT from a 3D perspective. Integration of epicardial and endocardial recordings during VT can be an useful strategy to infer about mid-myocardial activation (80). Tung and colleagues performed simultaneous endocardial and epicardial activation mapping and found that in activation patterns can occur on both myocardial surfaces (65). A 3D perspective of the VT circuit can enhance the precision of the ablative therapy and support a greater role for adjunctive strategies to address arrhythmias harbored in the mid-myocardium and subepicardium.

High density multipolar mapping systems allow for a collection of greater numbers of EGMs from smaller, more closely spaced electrodes during VT. Several animal and human studies have shown that one of the advantages of HD mapping is that far-field signals are significantly lessened by reducing the bipole spacing, improving near-field detection (81, 82). These HD catheters Advisor HD-Grid, Abbott, Abbott Park, Illinois; Optrell, Pentaray and Octaray, Biosense Webster, Diamond Bar, California; Orion, Boston Scientific, Marlborough, MA) can enhance mapping resolution in areas of low voltage and scar, enabling detection of areas of preserved myocardial fibers, and identification of diastolic electrograms during VT. Conventional catheters would likely classify these low voltage areas as dense scar. Mapping of the entire diastolic pathway, utilizing multielectrode mapping catheters, has been associated with a higher freedom from VT recurrence (83).

## Strategies to improve ablation

Radiofrequency ablation as an effective therapy for VT is based on the principle of delivering solid and durable lesions. One of the major determinants of lesion formation is an adequate contact between the catheter tip and the myocardial surface. One of the major technological advancements was the development of sensors at the distal tip capable of monitoring contact, contact force (CF) (84, 85). During VT ablation, a median contact force of 10 g within the scar zone has been shown to have the best correlation with effective lesion formation (86, 87). Although there is also evidence that contact force sensing catheters may not change long-term outcomes, it is important that we continue to explore markers of ablation efficacy (88).

In a certain subset of patients, especially those with mid-myocardial substrates, there is a growing need to develop new strategies for deeper lesion formation. Alternatives such as half-normal saline and dextrose solution are able to create larger lesions compared to saline irrigation (89). Modulating impedance is another strategy to augment lesion size, by increasing radiofrequency current using similar power settings. Clinically this can be achieved by placing additional surface dispersive electrode patches. However, it is important to remember that increasing current density with low-ionic irrigants, lowered impedance, and high power settings has the potential to increase the risk of complications, including steam pops.

**Figure 3:**
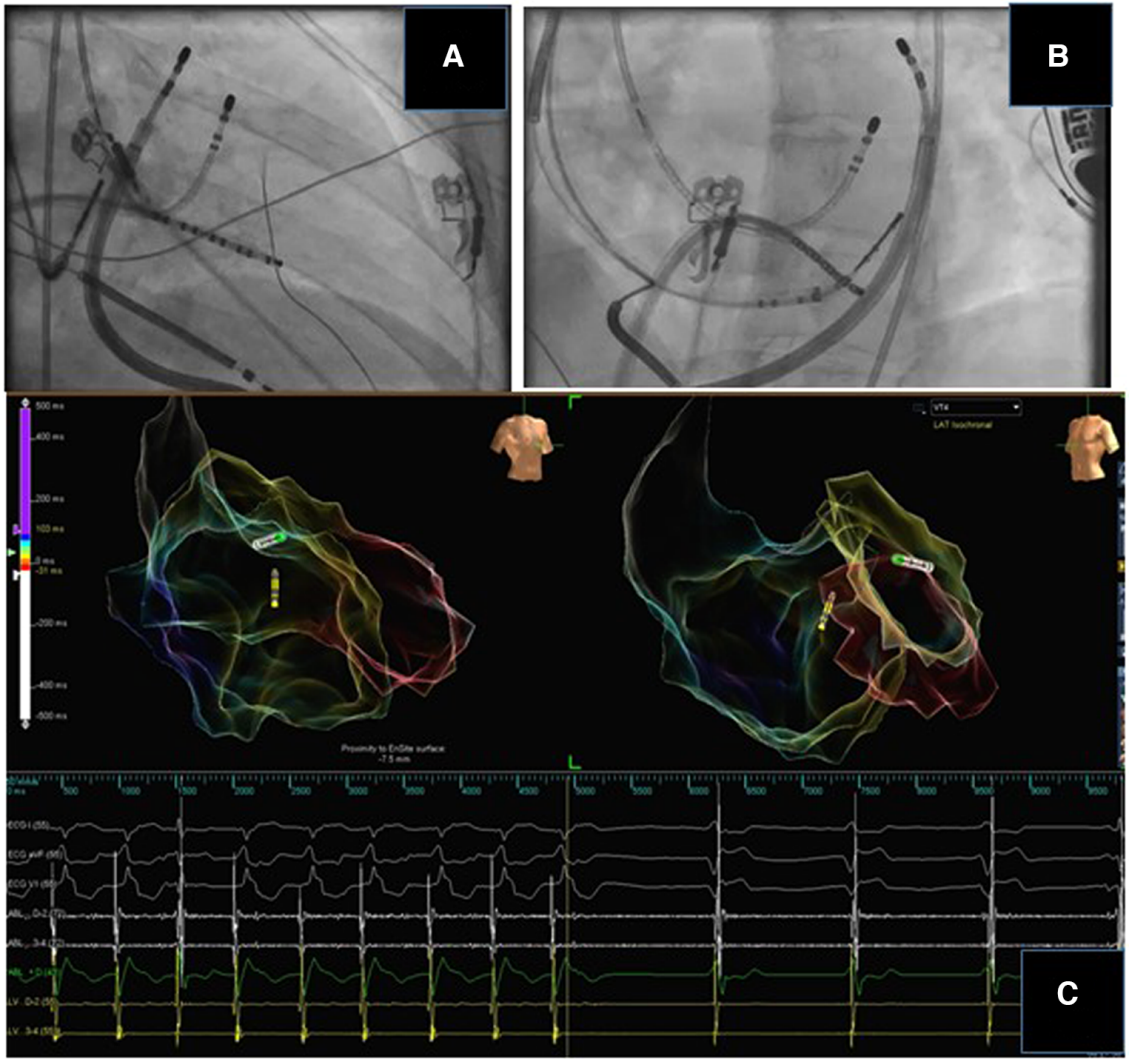
Bipolar ablation for intramural substrate. A case of cardiac sarcoidosis with an intramural substrate. Bipolar ablation was delivered through an endocardial and epicardial catheter at the basal lateral wall. The distance between the tips of the ablation catheters was 10 mm. There was termination of the tachycardia during ablation.

Utilizing two catheters for either simultaneous unipolar ablation or bipolar ablation can be used to achieve transmural lesions. Bipolar ablation uses two catheters connected to the radiofrequency generator (1 to the output terminal and 1 to the ground reference), placed on opposite surfaces of the myocardial tissues. This can also be achieved with simultaneous unipolar ablation with 2 catheters connected to separate radiofrequency generators. Observational case series have found these techniques to useful to terminate VT in patients who unsuccessful unipolar ablation of septal substrates (90) (Figure 3). Recently, another tool developed for deep lesion formation utilizes a catheter with an irrigated 27 gauge retractable needle-tipped electrode (91). This allows radiofrequency ablation to be delivered directly inside the myocardial wall, overcoming the issue of intramural lesion delivery. Remote magnetic navigation is an alternative to manual catheter control, and has shown to be a safe and feasible alternative (92).

**Figure 4:**
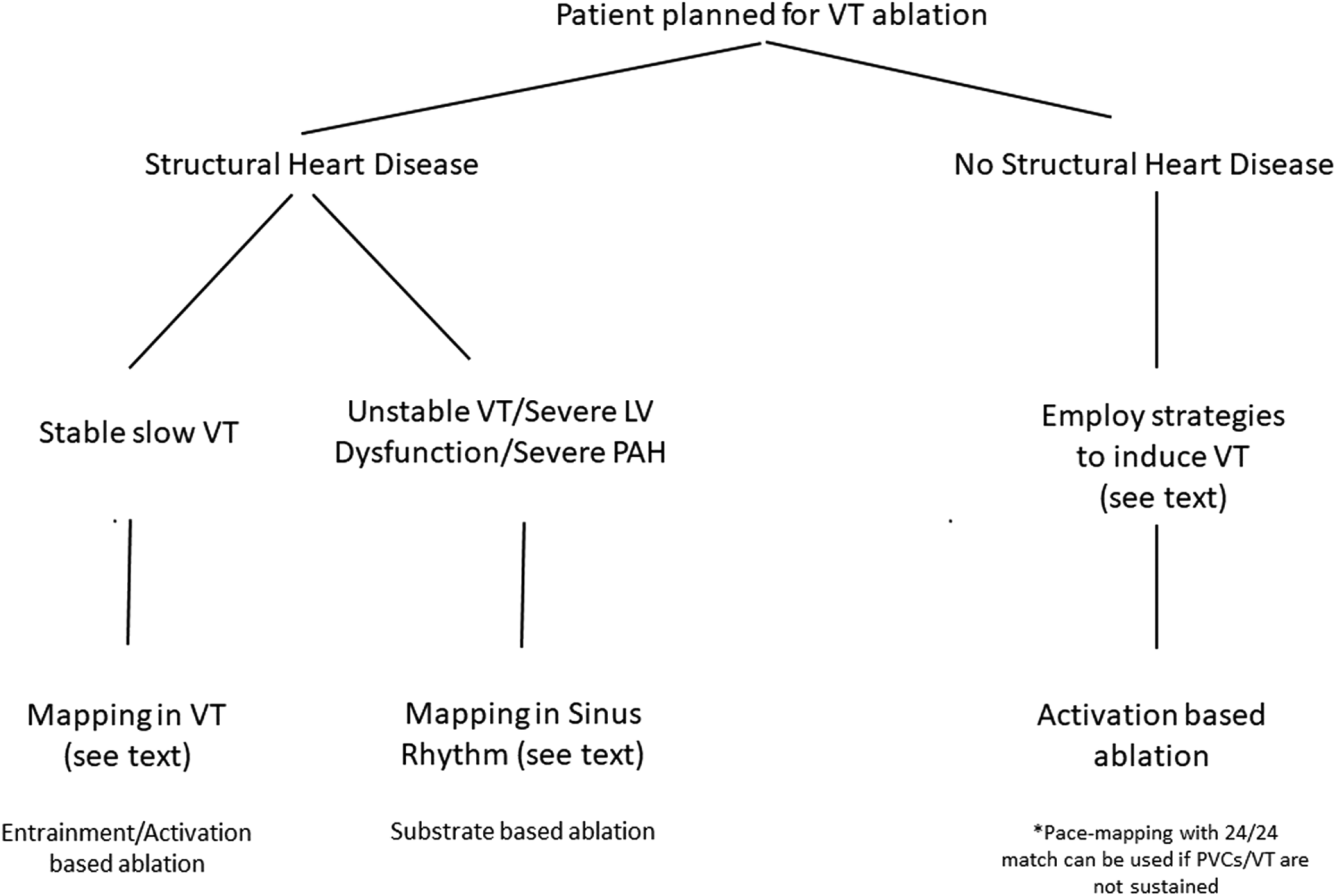
Summary of approach to VT ablation in patients with and without structural heart disease.

Identification of the optimal endpoints for VT ablation is crucial to improve the success rate of this procedure. The response to programmed electric stimulation at the end of the procedure has been traditionally used to evaluate not only the acute success but also to predict the long term outcome. The guidelines endorse noninducibility of PES as an endpoint for VT ablation (93). However, several studies have not been able to show a direct association between VT non-inducibility and long-term arrhythmia-free survival. Many potential confounders might affect VT inducibility including periprocedural antiarrhythmic drug therapy, type of anaesthesia, and heterogenous PES protocols. Santangeli and colleagues compared RV stimulation with LV stimulation within the scar in a series of 156 patients undergoing catheter ablation of post-infraction VT. RV stimulation induced clinical VTs in 31% of cases whereas stimulation within the scar induced clinical VTs in close to 70% of patients (93). The authors hypothesized that some RBBB VTs are best induced with lateral LV stimulation. Noninvasive PES (NIPS) has been used in some centres a few days after catheter ablation to identify patients at increased risk of VT recurrence. Studies suggest that performing NIPS a few days after the procedure should be strongly considered as an endpoint for catheter ablation of scar related VT (94).

Regarding substrate-based ablation approaches, there is an increasing need for novel endpoints beyond non-inducibility to evaluate the completeness of linear lesions and the elimination of abnormal electrograms. Studies that have assessed the role of late potential ablation in scar related VT have adopted procedural endpoints ranging from complete elimination of late potentials to failure to capture with high output pacing. Berruezo and colleagues also used elimination of all conductive channels and found that this method resulted in a very high rate of VT-free survival during median follow up of 11 months (95). A box lesion set approach has also been utilized to eliminate all potentially arrhythmogenic areas within the scar.

## Use of anticoagulation in the post-procedure period

There are limited data regarding the effectiveness and safety of post-ablation anticoagulation strategies after VT ablation. In the post op period, the benefits of anticoagulation need to be weighed against the risks of bleeding. Siontis and colleagues found that using a slowly escalating bridging regiment of UFH, followed by 3 months of oral anticoagulation is associated with low thromboembolic and bleeding risk after infract-related VT ablation (96). In the absence of extensive ablation, they found that antiplatelet therapy alone was a reasonable strategy. Recently, the STROKE-VT trial suggested that DOAC use following endocardial and/or epicardial ablation for left ventricular arrhythmia ablation was associated with a reduced risk of TIA or stroke (97). The risk of bleeding and thrombotic complications need to be considered and anticoagulation needs to be individualized.

## Conclusion

Safety and efficacy of catheter ablation for ventricular tachycardia has steadily improved. Better understanding of the pathophysiology of VT, advanced mapping systems as well as development of contact force sensing catheters have resulted in improved outcomes. Substrate modification strategies helps to safely and successfully ablate VTs in sick cohort of patients, as it eliminates the need for repeated induction of VT for mapping. Most importantly, utilizing a systematic approach to mapping and ablation of VT is crucial for management of these patients and improving overall clinical outcome.

## References

[1] Stevenson WG Wilber DJ Natale A Jackman WM Marchlinski FE Talbert T Irrigated radiofrequency catheter ablation guided by electroanatomic mapping for recurrent ventricular tachycardia after myocardial infarction: the multicenter thermocool ventricular tachycardia ablation trial. Circulation. (2008) 118:2773–82. 10.1161/CIRCULATIONAHA.108.788604 19064682

[2] Reddy VY Reynolds MR Neuzil P Richardson AW Taborsky M Jongnarangsin K Prophylactic catheter ablation for the prevention of defibrillator therapy. N Engl J Med. (2007) 357:2657–65. 10.1056/NEJMoa065457 18160685 PMC2390777

[3] Hutchinson MD Gerstenfeld EP Desjardins B Bala R Riley MP Garcia FC Endocardial unipolar voltage mapping to detect epicardial ventricular tachycardia substrate in patients with nonischemic left ventricular cardiomyopathy. Circ Arrhythm Electrophysiol. (2011) 4:49–55. 10.1161/CIRCEP.110.959957 21131557 PMC3041847

[4] Desjardins B Yokokawa M Good E Crawford T Latchamsetty R Jongnarangsin K Characteristics of intramural scar in patients with nonischemic cardiomyopathy and relation to intramural ventricular arrhythmias. Circ Arrhythm Electrophysiol. (2013) 6:891–7. 10.1161/CIRCEP.113.000073 23985383

[5] Sanchez JM Yuan C Hsia HH. Optimal ablation techniques for ventricular tachycardia management. J Innov Card Rhythm Manag. (2018) 9:2969–81. 10.19102/icrm.2018.090101 32477780 PMC7252666

[6] Cano O Hutchinson M Lin D Garcia F Zado E Bala R Electroanatomic substrate and ablation outcome for suspected epicardial ventricular tachycardia in left ventricular nonischemic cardiomyopathy. J Am Coll Cardiol. (2009) 54:799–808. 10.1016/j.jacc.2009.05.032 19695457

[7] Soejima K Stevenson WG Sapp JL Selwyn AP Couper G Epstein LM. Endocardial and epicardial radiofrequency ablation of ventricular tachycardia associated with dilated cardiomyopathy: the importance of low-voltage scars. J Am Coll Cardiol. (2004) 43:1834–42. 10.1016/j.jacc.2004.01.029 15145109

[8] Campbell T Bennett RG Kotake Y Kumar S. Updates in ventricular tachycardia ablation. Korean Circ J. (2021) 51:15–42. 10.4070/kcj.2020.0436 33377327 PMC7779814

[9] Ratheendran AC Subramanian M Bhanu DK Prabhu MA Kannan R Natarajan KU Fragmented QRS on electrocardiography as a predictor of myocardial scar in patients with hypertrophic cardiomyopathy. Acta Cardiol. (2020) 75:42–6. 10.1080/00015385.2018.1547355 30602338

[10] Miller JM Marchlinski FE Buxton AE Josephson ME. Relationship between the 12-lead electrocardiogram during ventricular tachycardia and endocardial site of origin in patients with coronary artery disease. Circulation. (1988) 77:759–66. 10.1161/01.CIR.77.4.759 3349580

[11] Sapp JL Bar-Tal M Howes AJ Toma JE El-Damaty A Warren JW Real-time localization of ventricular tachycardia origin from the 12-lead electrocardiogram. JACC Clin Electrophysiol. (2017) 3:687–99. 10.1016/j.jacep.2017.02.024 29759537

[12] Zhou S AbdelWahab A Horacek BM MacInnis PJ Warren JW Davis JS Prospective assessment of an automated intraprocedural 12-lead ECG-based system for localization of early left ventricular activation. Circ Arrhythm Electrophysiol. (2020) 13:e008262. 10.1161/CIRCEP.119.008262 32538133 PMC7375941

[13] Valles E Bazan V Marchlinski FE. ECG criteria to identify epicardial ventricular tachycardia in nonischemic cardiomyopathy. Circ Arrhythm Electrophysiol. (2010) 3:63–71. 10.1161/CIRCEP.109.859942 20008307

[14] Martinek M Stevenson WG Inada K Tokuda M Tedrow UB. QRS characteristics fail to reliably identify ventricular tachycardias that require epicardial ablation in ischemic heart disease. J Cardiovasc Electrophysiol. (2012) 23:188–93. 10.1111/j.1540-8167.2011.02179.x 21955120

[15] Panda S Kaur D Lalukota K Sundar G Pavri BB Narasimhan C. Pleomorphism during ventricular tachycardia: a distinguishing feature between cardiac sarcoidosis and idiopathic VT. Pacing Clin Electrophysiol. (2015) 38:694–9. 10.1111/pace.12626 25754130

[16] Hocini M Shah AJ Pascale P Roten L Komatsu Y Daly M Body surface electrocardiographic mapping for non-invasive identification of arrhythmic sources. Arrhythm Electrophysiol Rev. (2013) 2:16–22. 10.15420/aer.2013.2.1.16 26835035 PMC4711575

[17] Desjardins B Crawford T Good E Oral H Chugh A Pelosi F Infarct architecture and characteristics on delayed enhanced magnetic resonance imaging and electroanatomic mapping in patients with postinfarction ventricular arrhythmia. Heart Rhythm. (2009) 6:644–51. 10.1016/j.hrthm.2009.02.018 19389653 PMC2735883

[18] Bogun FM Desjardins B Good E Gupta S Crawford T Oral H Delayed-enhanced magnetic resonance imaging in nonischemic cardiomyopathy: utility for identifying the ventricular arrhythmia substrate. J Am Coll Cardiol. (2009) 53:1138–45. 10.1016/j.jacc.2008.11.052 19324259 PMC2747602

[19] Kuo L Liang JJ Nazarian S Marchlinski FE. Multimodality imaging to guide ventricular tachycardia ablation in patients with non-ischaemic cardiomyopathy. Arrhythm Electrophysiol Rev. (2020) 8:255–64. 10.15420/aer.2019.37.3 32685156 PMC7358957

[20] Kuo L Liang JJ Han Y Frankel DS Santangeli P Callans DJ Association of septal late gadolinium enhancement on cardiac magnetic resonance with ventricular tachycardia ablation targets in nonischemic cardiomyopathy. J Cardiovasc Electrophysiol. (2020) 31:3262–76. 10.1111/jce.14777 33070414

[21] Piers SR Tao Q de Riva Silva M Siebelink HM Schalij MJ van der Geest RJ CMR-based identification of critical isthmus sites of ischemic and nonischemic ventricular tachycardia. JACC Cardiovasc Imaging. (2014) 7:774–84. 10.1016/j.jcmg.2014.03.013 25051947

[22] Fernandez-Armenta J Berruezo A Andreu D Camara Os Silva E Serra L Three-dimensional architecture of scar and conducting channels based on high resolution ce-CMR: insights for ventricular tachycardia ablation. Circ Arrhythm Electrophysiol. (2013) 6:528–37. 10.1161/CIRCEP.113.000264 23685537

[23] Rashid S Rapacchi S Shivkumar K Plotnik A Finn JP Hu P. Modified wideband three-dimensional late gadolinium enhancement MRI for patients with implantable cardiac devices. Magn Reson Med. (2016) 75:572–84. 10.1002/mrm.25601 25772155 PMC4661130

[24] Ibrahim EH Runge M Stojanovska J Agarwal P Ghadimi-Mahani M Attili A Optimized cardiac magnetic resonance imaging inversion recovery sequence for metal artifact reduction and accurate myocardial scar assessment in patients with cardiac implantable electronic devices. World J Radiol. (2018) 10:100–7. 10.4329/wjr.v10.i9.100 30310544 PMC6177559

[25] Mahida S Sacher F Dubois R Sermesant M Bogun F Haïssaguerre M Cardiac imaging in patients with ventricular tachycardia. Circulation. (2017) 136:2491–507. 10.1161/CIRCULATIONAHA.117.029349 29255125

[26] Tian J Jeudy J Smith MF Jimenez A Yin X Bruce PA Three-dimensional contrast-enhanced multidetector CT for anatomic, dynamic, and perfusion characterization of abnormal myocardium to guide ventricular tachycardia ablations. Circ Arrhythm Electrophysiol. (2010) 3:496–504. 10.1161/CIRCEP.109.889311 20657032

[27] Ghannam M Cochet H Jais P Sermesant M Patel S Siontis KC Correlation between computer tomography-derived scar topography and critical ablation sites in postinfarction ventricular tachycardia. J Cardiovasc Electrophysiol. (2018) 29:438–45. 10.1111/jce.13441 29380921

[28] Takigawa M Duchateau J Sacher F Martin R Vlachos K Kitamura T Are wall thickness channels defined by computed tomography predictive of isthmuses of postinfarction ventricular tachycardia? Heart Rhythm. (2019) 16:1661–8. 10.1016/j.hrthm.2019.06.012 31207315

[29] Yamashita S Sacher F Mahida S Berte B Lim HS Komatsu Y Image integration to guide catheter ablation in scar-related ventricular tachycardia. J Cardiovasc Electrophysiol. (2016) 27:699–708. 10.1111/jce.12963 26918883

[30] Esposito A Palmisano A Antunes S Maccabelli G Colantoni C Rancoita PMV Cardiac CT with delayed enhancement in the characterization of ventricular tachycardia structural substrate: relationship between CT-segmented scar and electro-anatomic mapping. JACC Cardiovasc Imaging. (2016) 9:822–32. 10.1016/j.jcmg.2015.10.024 26897692

[31] Cochet H Komatsu Y Sacher F Jadidi AS Scherr D Riffaud M Integration of merged delayed-enhanced magnetic resonance imaging and multidetector computed tomography for the guidance of ventricular tachycardia ablation: a pilot study. J Cardiovasc Electrophysiol. (2013) 24:419–26. 10.1111/jce.12052 23252727

[32] Kaur D Roukoz H Shah M Yalagudri S Pandurangi U Chennapragada S Impact of the inflammation on the outcomes of catheter ablation of drug-refractory ventricular tachycardia in cardiac sarcoidosis. J Cardiovasc Electrophysiol. (2020) 31:612–20. 10.1111/jce.14341 31916658

[33] Cheung E Ahmad S Aitken M Chan R Iwanochko RM Balter M Combined simultaneous FDG-PET/MRI with T1 and T2 mapping as an imaging biomarker for the diagnosis and prognosis of suspected cardiac sarcoidosis. Eur J Hybrid Imaging. (2021) 5:24. 10.1186/s41824-021-00119-w 34913098 PMC8674394

[34] Della Bella P Brugada J Zeppenfeld K Merino J Neuzil P Maury P Epicardial ablation for ventricular tachycardia: a European multicenter study. Circ Arrhythm Electrophysiol. (2011) 4:653–9. 10.1161/CIRCEP.111.962217 21841191

[35] Sacher F Roberts-Thomson K Maury P Tedrow U Nault I Steven D Epicardial ventricular tachycardia ablation a multicenter safety study. J Am Coll Cardiol. (2010) 55:2366–72. 10.1016/j.jacc.2009.10.084 20488308

[36] Subramanian M Ravilla VV Yalagudri S Saggu DK Rangaswamy VV d'Avila A CT-guided percutaneous epicardial access for ventricular tachycardia ablation: a proof-of-concept study. J Cardiovasc Electrophysiol. (2021) 32:2665–72. 10.1111/jce.15210 34405472

[37] Kupo P Pap R Saghy L Tényi D Bálint A Debreceni D Ultrasound guidance for femoral venous access in electrophysiology procedures-systematic review and meta-analysis. J Interv Card Electrophysiol. (2020) 59:407–14. 10.1007/s10840-019-00683-z 31823233 PMC7591449

[38] Sobolev M Shiloh AL Di Biase L Slovut DP. Ultrasound-guided cannulation of the femoral vein in electrophysiological procedures: a systematic review and meta-analysis. Europace. (2017) 19:850–5. 10.1093/europace/euw113 27207813

[39] Deng Y Naeini PS Razavi M Collard CD Tolpin DA Anton JM. Anesthetic management in radiofrequency catheter ablation of ventricular tachycardia. Tex Heart Inst J. (2016) 43:496–502. 10.14503/THIJ-15-5688 28100967 PMC5179153

[40] Santangeli P Frankel DS Tung R Vaseghi M Sauer WH Tzou WS Early mortality after catheter ablation of ventricular tachycardia in patients with structural heart disease. J Am Coll Cardiol. (2017) 69:2105–15. 10.1016/j.jacc.2017.02.044 28449770

[41] Nof E Stevenson WG John RM. Catheter ablation for ventricular arrhythmias. Arrhythm Electrophysiol Rev. (2013) 2:45–52. 10.15420/aer.2013.2.1.45 26835040 PMC4711562

[42] Wijnmaalen AP van der Geest RJ van Huls van Taxis CFB Siebelink HMJ Kroft LJM Bax JJ Head-to-head comparison of contrast-enhanced magnetic resonance imaging and electroanatomical voltage mapping to assess post-infarct scar characteristics in patients with ventricular tachycardias: real-time image integration and reversed registration. Eur Heart J. (2011) 32:104–14. 10.1093/eurheartj/ehq345 20864488

[43] Bunch TJ Weiss JP Crandall BG Day JD Dimarco JP Ferguson JD Image integration using intracardiac ultrasound and 3D reconstruction for scar mapping and ablation of ventricular tachycardia. J Cardiovasc Electrophysiol. (2010) 21:678–84. 10.1111/j.1540-8167.2009.01680.x 20102427

[44] Khaykin Y Klemm O Verma A. First human experience with real-time integration of intracardiac echocardiography and 3D electroanatomical imaging to guide right free wall accessory pathway ablation. Europace. (2008) 10:116–7. 10.1093/europace/eum243 18003632

[45] Bauer BK Meier C Bietenbeck M Lange PS Eckardt L Yilmaz A. Cardiovascular magnetic resonance-guided radiofrequency ablation: where are we now? JACC Clin Electrophysiol. (2022) 8:261–74. 10.1016/j.jacep.2021.11.017 35210090

[46] Sticherling C Marin F Birnie D Boriani G Calkins H Dan GA Antithrombotic management in patients undergoing electrophysiological procedures: a European heart rhythm association (EHRA) position document endorsed by the ESC working group thrombosis, heart rhythm society (HRS), and Asia pacific heart rhythm society (APHRS). Europace. (2015) 17:1197–214. 10.1093/europace/euv190 26105732

[47] Tschabrunn CM Zado ES Schaller RD Garcia FC Kumareswaran R Hsue W Isolated critical epicardial arrhythmogenic substrate abnormalities in patients with arrhythmogenic right ventricular cardiomyopathy and ventricular tachycardia. Heart Rhythm. (2022) 19:538–45. 10.1016/j.hrthm.2021.11.035 34883271

[48] Marchlinski FE Callans DJ Gottlieb CD Zado E. Linear ablation lesions for control of unmappable ventricular tachycardia in patients with ischemic and nonischemic cardiomyopathy. Circulation. (2000) 101:1288–96. 10.1161/01.CIR.101.11.1288 10725289

[49] Kettering K Weig HJ Reimold M Schwegler AC Busch M Laszlo R Catheter ablation of ventricular tachycardias in patients with ischemic cardiomyopathy: validation of voltage mapping criteria for substrate modification by myocardial viability assessment using FDG PET. Clin Res Cardiol. (2010) 99:753–60. 10.1007/s00392-010-0182-2 20532538

[50] Arenal A del Castillo S Gonzalez-Torrecilla E Atienza F Ortiz M Jimenez J Tachycardia-related channel in the scar tissue in patients with sustained monomorphic ventricular tachycardias: influence of the voltage scar definition. Circulation. (2004) 110:2568–74. 10.1161/01.CIR.0000145544.35565.47 15492309

[51] Hsia HH Lin D Sauer WH Callans DJ Marchlinski FE. Anatomic characterization of endocardial substrate for hemodynamically stable reentrant ventricular tachycardia: identification of endocardial conducting channels. Heart Rhythm. (2006) 3:503–12. 10.1016/j.hrthm.2006.01.015 16648052

[52] Mountantonakis SE Park RE Frankel DS Dixit S Cooper J Callans D Relationship between voltage map “channels” and the location of critical isthmus sites in patients with post-infarction cardiomyopathy and ventricular tachycardia. J Am Coll Cardiol. (2013) 61:2088–95. 10.1016/j.jacc.2013.02.031 23524215

[53] Spears DA Suszko AM Dalvi R Crean AM Ivanov J Nanthakumar K Relationship of bipolar and unipolar electrogram voltage to scar transmurality and composition derived by magnetic resonance imaging in patients with nonischemic cardiomyopathy undergoing VT ablation. Heart Rhythm. (2012) 9:1837–46. 10.1016/j.hrthm.2012.07.022 22846338

[54] Venlet J Piers SRD Kapel GFL de Riva M Pauli PFG van der Geest RJ Unipolar endocardial voltage mapping in the right ventricle: optimal cutoff values correcting for computed tomography-derived epicardial fat thickness and their clinical value for substrate delineation. Circ Arrhythm Electrophysiol. (2017) 10:e005175. 10.1161/CIRCEP.117.005175 28798020

[55] Qian PC Oberfeld B Schaeffer B Nakamura T John RM Sapp JL Frequency content of unipolar electrograms may predict deep intramural excitable substrate: insights from intramural needle catheter ablation of ventricular tachycardia. JACC Clin Electrophysiol. (2020) 6:760–9. 10.1016/j.jacep.2020.03.003 32703556

[56] Fast VG Kleber AG. Role of wavefront curvature in propagation of cardiac impulse. Cardiovasc Res. (1997) 33:258–71. 10.1016/S0008-6363(96)00216-7 9074688

[57] Jais P Maury P Khairy P Sacher F Nault I Komatsu Y Elimination of local abnormal ventricular activities: a new end point for substrate modification in patients with scar-related ventricular tachycardia. Circulation. (2012) 125:2184–96. 10.1161/CIRCULATIONAHA.111.043216 22492578

[58] Komatsu Y. Substrate-based approach for ventricular tachycardia in structural heart disease: tips for mapping and ablation. J Arrhythm. (2014) 30:272–82. 10.1016/j.joa.2014.04.014

[59] Bogun F Good E Reich S Elmouchi D Igic P Lemola K Isolated potentials during sinus rhythm and pace-mapping within scars as guides for ablation of post-infarction ventricular tachycardia. J Am Coll Cardiol. (2006) 47:2013–9. 10.1016/j.jacc.2005.12.062 16697318

[60] Ciaccio EJ Tosti AC Scheinman MM. Method to predict isthmus location in ventricular tachycardia caused by reentry with a double-loop pattern. J Cardiovasc Electrophysiol. (2005) 16:528–36. 10.1046/j.1540-8167.2005.40638.x 15877625

[61] Irie T Yu R Bradfield JS Vaseghi M Buch EF Ajijola O Relationship between sinus rhythm late activation zones and critical sites for scar-related ventricular tachycardia: systematic analysis of isochronal late activation mapping. Circ Arrhythm Electrophysiol. (2015) 8:390–9. 10.1161/CIRCEP.114.002637 25740836 PMC4695215

[62] Anter E Kleber AG Rottmann M Leshem E Barkagan M Tschabrunn CM Infarct-related ventricular tachycardia: redefining the electrophysiological substrate of the isthmus during sinus rhythm. JACC Clin Electrophysiol. (2018) 4:1033–48. 10.1016/j.jacep.2018.04.007 30139485

[63] Nayyar S Downar E Bhaskaran AP Masse S Nanthakumar K. Signature signal strategy: electrogram-based ventricular tachycardia mapping. Heart Rhythm. (2020) 17:2000–9. 10.1016/j.hrthm.2020.06.022 32590152

[64] Kleber AG Rudy Y. Basic mechanisms of cardiac impulse propagation and associated arrhythmias. Physiol Rev. (2004) 84:431–88. 10.1152/physrev.00025.2003 15044680

[65] Aziz Z Shatz D Raiman M Upadhyay GA Beaser AD Besser SA Targeted ablation of ventricular tachycardia guided by wavefront discontinuities during sinus rhythm: a new functional substrate mapping strategy. Circulation. (2019) 140:1383–97. 10.1161/CIRCULATIONAHA.119.042423 31533463

[66] Porta-Sanchez A Jackson N Lukac P Kristiansen SB Nielsen JM Gizurarson S Multicenter study of ischemic ventricular tachycardia ablation with decrement-evoked potential (DEEP) mapping with extra stimulus. JACC Clin Electrophysiol. (2018) 4:307–15. 10.1016/j.jacep.2017.12.005 30089555

[67] Di Biase L Santangeli P Burkhardt DJ Bai R Mohanty P Carbucicchio C Endo-epicardial homogenization of the scar versus limited substrate ablation for the treatment of electrical storms in patients with ischemic cardiomyopathy. J Am Coll Cardiol. (2012) 60:132–41. 10.1016/j.jacc.2012.03.044 22766340

[68] Vazquez-Calvo S Garre P Sanchez-Somonte P Borras R Quinto L Caixal G Orthogonal high-density mapping with ventricular tachycardia isthmus analysis vs. pure substrate ventricular tachycardia ablation: a case-control study. Front Cardiovasc Med. (2022) 9:912335. 10.3389/fcvm.2022.912335 35979023 PMC9376368

[69] Tschabrunn CM Roujol S Nezafat R Faulkner-Jones B Buxton AE Josephson ME A swine model of infarct-related reentrant ventricular tachycardia: electroanatomic, magnetic resonance, and histopathological characterization. Heart Rhythm. (2016) 13:262–73. 10.1016/j.hrthm.2015.07.030 26226214 PMC4747106

[70] Tschabrunn CM Roujol S Dorman NC Nezafat R Josephson ME Anter E. High-resolution mapping of ventricular scar: comparison between single and multielectrode catheters. Circ Arrhythm Electrophysiol. (2016) 9(6). 10.1161/CIRCEP.115.003841 27307518 PMC4911826

[71] Stevenson WG Sager PT Natterson PD Saxon LA Middlekauff HR Wiener I. Relation of pace mapping QRS configuration and conduction delay to ventricular tachycardia reentry circuits in human infarct scars. J Am Coll Cardiol. (1995) 26:481–8. 10.1016/0735-1097(95)80026-D 7608454

[72] de Chillou C Groben L Magnin-Poull I Andronache M Abbas MM Zhang N Localizing the critical isthmus of postinfarct ventricular tachycardia: the value of pace-mapping during sinus rhythm. Heart Rhythm. (2014) 11:175–81. 10.1016/j.hrthm.2013.10.042 24513915

[73] Wissner E Menon SY Metzner A Schoonderwoerd B Nuyens D Makimoto H Long-term outcome after catheter ablation for left posterior fascicular ventricular tachycardia without development of left posterior fascicular block. J Cardiovasc Electrophysiol. (2012) 23:1179–84. 10.1111/j.1540-8167.2012.02377.x 22697499

[74] Gopi A Nair SG Shelke A Saggu DK Yalagudri S Reddy P A stepwise approach to the induction of idiopathic fascicular ventricular tachycardia. J Interv Card Electrophysiol. (2015) 44:17–22. 10.1007/s10840-015-0022-4 26139310

[75] Nazer B Woods C Dewland T Moyers B Badhwar N Gerstenfeld EP. Importance of ventricular tachycardia induction and mapping for patients referred for epicardial ablation. Pacing Clin Electrophysiol. (2015) 38:1333–42. 10.1111/pace.12703 26228002

[76] Stevenson WG Weiss JN Wiener I Nademanee K Wohlgelernter D Yeatman L Resetting of ventricular tachycardia: implications for localizing the area of slow conduction. J Am Coll Cardiol. (1988) 11:522–9. 10.1016/0735-1097(88)91526-4 2449482

[77] Waldo AL Henthorn RW. Use of transient entrainment during ventricular tachycardia to localize a critical area in the reentry circuit for ablation. Pacing Clin Electrophysiol. (1989) 12:231–44. 10.1111/j.1540-8159.1989.tb02652.x 2466258

[78] Tung R. Challenges and pitfalls of entrainment mapping of ventricular tachycardia: ten illustrative concepts. Circ Arrhythm Electrophysiol. (2017) 10:e004560. 10.1161/CIRCEP.116.004560 28408650

[79] Soejima K Stevenson WG Maisel WH Delacretaz E Brunckhorst CB Ellison KE The *N* + 1 difference: a new measure for entrainment mapping. J Am Coll Cardiol. (2001) 37:1386–94. 10.1016/S0735-1097(01)01163-9 11300451

[80] Tung R Raiman M Liao H Zhan X Chung FP Nagel R Simultaneous endocardial and epicardial delineation of 3D reentrant ventricular tachycardia. J Am Coll Cardiol. (2020) 75:884–97. 10.1016/j.jacc.2019.12.044 32130924

[81] Jiang R Beaser AD Aziz Z Upadhyay GA Nayak HM Tung R. High-density grid catheter for detailed mapping of sinus rhythm and scar-related ventricular tachycardia: comparison with a linear duodecapolar catheter. JACC Clin Electrophysiol. (2020) 6:311–23. 10.1016/j.jacep.2019.11.007 32192682

[82] Cheung JW. Targeting abnormal electrograms for substrate-based ablation of ventricular tachycardia: can we ablate smarter? JACC Clin Electrophysiol. (2020) 6:812–4. 10.1016/j.jacep.2020.04.013 32703563

[83] Hadjis A Frontera A Limite LR Bisceglia C Bognoni L Foppoli L Complete electroanatomic imaging of the diastolic pathway is associated with improved freedom from ventricular tachycardia recurrence. Circ Arrhythm Electrophysiol. (2020) 13:e008651. 10.1161/CIRCEP.120.008651 32755381 PMC7495983

[84] Mizuno H Vergara P Maccabelli G Trevisi N Eng SC Brombin C Contact force monitoring for cardiac mapping in patients with ventricular tachycardia. J Cardiovasc Electrophysiol. (2013) 24:519–24. 10.1111/jce.12080 23373693

[85] Elsokkari I Sapp JL Doucette S Parkash R Gray CJ Gardner MJ Role of contact force in ischemic scar-related ventricular tachycardia ablation; optimal force required and impact of left ventricular access route. J Interv Card Electrophysiol. (2018) 53:323–31. 10.1007/s10840-018-0396-1 29946899

[86] Zhao Z Liu X Gao L Xi Y Chen Q Chang D Benefit of contact force-guided catheter ablation for treating premature ventricular contractions. Tex Heart Inst J. (2020) 47:3–9. 10.14503/THIJ-17-6441 32148445 PMC7046358

[87] Jesel L Sacher F Komatsu Y Daly M Zellerhoff S Lim HS Characterization of contact force during endocardial and epicardial ventricular mapping. Circ Arrhythm Electrophysiol. (2014) 7:1168–73. 10.1161/CIRCEP.113.001219 25258362

[88] Elbatran AI Li A Gallagher MM Kaba R Norman M Behr ER Contact force sensing in ablation of ventricular arrhythmias using a 56-hole open-irrigation catheter: a propensity-matched analysis. J Interv Card Electrophysiol. (2021) 60:543–53. 10.1007/s10840-020-00756-4 32440943 PMC8134314

[89] Nguyen DT Gerstenfeld EP Tzou WS Jurgens PT Zheng L Schuller J Radiofrequency ablation using an open irrigated electrode cooled with half-normal saline. JACC Clin Electrophysiol. (2017) 3:1103–10. 10.1016/j.jacep.2017.03.006 29759492

[90] Della Bella P Peretto G Paglino G Bisceglia C Radinovic A Sala S Bipolar radiofrequency ablation for ventricular tachycardias originating from the interventricular septum: safety and efficacy in a pilot cohort study. Heart Rhythm. (2020) 17:2111–8. 10.1016/j.hrthm.2020.06.025 32599177

[91] Sapp JL Beeckler C Pike R Parkash R Gray CJ Zeppenfeld K Initial human feasibility of infusion needle catheter ablation for refractory ventricular tachycardia. Circulation. (2013) 128:2289–95. 10.1161/CIRCULATIONAHA.113.003423 24036605

[92] Aryana A d'Avila A Heist EK Mela T Singh JP Ruskin JN Remote magnetic navigation to guide endocardial and epicardial catheter mapping of scar-related ventricular tachycardia. Circulation. (2007) 115:1191–200. 10.1161/CIRCULATIONAHA.106.672162 17296855

[93] Santangeli P Frankel DS Marchlinski FE. End points for ablation of scar-related ventricular tachycardia. Circ Arrhythm Electrophysiol. (2014) 7:949–60. 10.1161/CIRCEP.114.001585 25336365

[94] Frankel DS Mountantonakis SE Zado ES Anter E Bala R Cooper JM Noninvasive programmed ventricular stimulation early after ventricular tachycardia ablation to predict risk of late recurrence. J Am Coll Cardiol. (2012) 59:1529–35. 10.1016/j.jacc.2012.01.026 22516442

[95] Berruezo A Fernandez-Armenta J Mont L Zeljko H Andreu D Herczku C Combined endocardial and epicardial catheter ablation in arrhythmogenic right ventricular dysplasia incorporating scar dechanneling technique. Circ Arrhythm Electrophysiol. (2012) 5:111–21. 10.1161/CIRCEP.110.960740 22205683

[96] Siontis KC Jame S Sharaf Dabbagh G Latchamsetty R Jongnarangsin K Morady F Thromboembolic prophylaxis protocol with warfarin after radiofrequency catheter ablation of infarct-related ventricular tachycardia. J Cardiovasc Electrophysiol. (2018) 29:584–90. 10.1111/jce.13418 29315941

[97] Lakkireddy D Shenthar J Garg J Padmanabhan D Gopinathannair R Di Biase L Safety/efficacy of DOAC versus aspirin for reduction of risk of cerebrovascular events following VT ablation. JACC Clin Electrophysiol. (2021) 7:1493–501. 10.1016/j.jacep.2021.07.010 34393085

